# The relevance of educational attainments of parents of medical students for health workforce planning: data from Guiné-Bissau

**DOI:** 10.1186/s12960-020-00528-1

**Published:** 2020-11-25

**Authors:** Paulo Ferrinho, Inês Fronteira, Tiago Correia, Clotilde Neves

**Affiliations:** 1 Global Health and Tropical Medicine Research Center, Instituto de Higiene e Medicina Tropical, Universidade Nova de Lisboa, Rua da Junqueira 100, 1349 008 Alcântara, Lisbon, Portugal; 2 Ministério da Saúde, Bissau, Guinea-Bissau

**Keywords:** Guiné-Bissau health workforce planning, Level of education of parents, Medical students, Social reproduction of gender inequality

## Abstract

**Background:**

In this article, we analyze data collected in the context of health workforce planning (HWFP) for Guiné-Bissau as part of the development of the third National Health Strategy, to study the relationship between educational achievement of parents and medical student characteristics and professional expectations.

**Methods:**

Cross-sectional analytical study of all first-year medical students in Guiné-Bissau during December 2016.

**Results:**

Our results confirm that the isolated effect of each parent is different as it is the combined education of both parents. Parental influence also seems to vary according to the sex of the offspring. The higher the education of the father, the stronger the urban background of the offspring. Level of education of parents is also important in relation to the decision to study medicine and the age of starting those studies. It is also an important influence as to expectation regarding place of future practice: the highest the educational level, particularly of the father, the highest the expectation for a future urban practice.

**Conclusions:**

Our main interest in medical education is to study it as a health system intervention in order to contribute to health system’s strengthening in fragile states. This is discussed in the context of two frameworks: the labor market framework and WHO’s health system strengthening framework. Our data and that of others, recognize that household characteristics are important regarding future training and a future career in the health sector. This recognition should be integrated into HWFP frameworks.

## Background

Education and its multiple effects in policies, institutions and behaviors has long been a topic of sociological inquiry [1, 2]. These inquiries have shown a positive link between socio-economic status (SES) and children’s educational achievements [3, 4]. The literature further suggests a strong correlation between this mediation and the education attained by parents as a clear sign of the processes of social reproduction [4, 6–21]. This is true for the most diverse cultural contexts and has been confirmed from studies in Africa [11, 22–26]. This influence extends to college attendance and academic success at university level [19, 27].

Some literature, sometimes conflicting, also differentiates between the influence of fathers and mothers, which resonates on broader issues of gender and parental roles [8, 13, 15, 20, 21, 28]. Furthermore, the exact mechanisms whereby the level of parental education influences their child’s educational outcomes are not always clear. It has been argued on the basis of genetics, association with level of material resources, support for school work, occupation of the parents, or the transmission of cultural capital, i.e., “the behaviors that are rewarded in the school environment” [8, 11].

Regardless of how these mechanisms operate in specific social groups and countries, the influence of parent’s educational background on students’ scholar performance is of importance in low-income countries in order to develop policies to tackle this issue.

In this article, we analyze data collected in the context of health workforce planning (HWFP) for Guiné-Bissau as part of the development of the third National Health Strategy [29], to study the relationship between educational achievement of parents and medical student characteristics and professional expectations.

## Methods

A number of previous studies have been published about students attending the Cuba-supported Faculdade de Medicina Raúl Díaz-Argüelles García [30, 31]. In December 2016, in a context of HWFP and health strategy development, a censitary study [32] was done among the 108 first-year students attending the same faculty [33].

Besides collecting data on the variables studied in a previous survey, as reported elsewhere [30, 31], we also collected data on the level of education of the father and of the mother of each student. Data was recorded into three variables: education of the father and education of the mother with three categories each (parent did not complete secondary education; parent with complete secondary education; and parent with some level of complete post-secondary education) and combined parental education (one of the parents did not complete secondary education, one of the parents did not complete post-secondary education, both parents have some level of complete post-secondary education). No “complete secondary education” included not having any education, having complete or incomplete primary education or incomplete secondary education; no “complete post-secondary education” included complete secondary education, incomplete or complete technical education or incomplete post-graduate university studies, incomplete BA or incomplete licenciatura. “Some level of complete post-secondary education” included complete post-graduate university studies, Ba, licenciatura, Master, or doctorate or incomplete master or incomplete doctorate.

Statistical analysis was carried out using IBM SPSS 25. The dependent variables were cross-tabulated with sex; place of birth; place of primary education; place of secondary education; marital status; responsibility for dependents; working students; family influence on decision to become a doctor; place of future practice (capital city vs elsewhere); level of future practice (hospital vs health center); and type of future practice (public vs private). Statistical significance for cross-tabulation of categorical variables was tested by Pearson Chi-square. For age and age of decision to study, central tendency and dispersion measures were computed and significance of differences tested with ANOVA [34].

## Results

The most frequent among medical students was for the mother not to have complete secondary education, while fathers tended to have some levels of complete secondary or post-secondary education. The majority of students had at least one parent who had not complete secondary education. Fathers tend to be more educated than mothers (Table 1).

**Table 1:** Distribution of educational achievement of father, mother and combined

	Education of mother	Education of father	Combined level of education of father and mother
Parent did not complete secondary education	53 (58%)	31 (34%)	–
Parent with complete secondary education	15(16%)	18 (20%)	–
Parent with some level of complete post-secondary education	24 (26%)	43 (46%)	–
One of the parents did not complete secondary education	–	–	54 (61%)
One of the parents did not complete post-secondary education	–	–	13 (15%)
Both parents have some level of complete post-secondary education	–	–	21 (24%)

Female students had a higher percentage of fathers and mothers with tertiary education than males (Table 2).

**Table 2:** Distribution of education of parents per sex of students (column %)

Education of father	Sex of the student^*^		Education of mother	Sex of the student^**^		Combined education of both parents	Sex of the student^***^	
	M (*n* = 66)	F (*n* = 26)		M (*n* = 66)	F (*n* = 26)		M (*n* = 62)	F (*n* = 26)
Father did not complete secondary education	42.4% *N* = 28	11.5% *N* = 3	Mother did not complete secondary education	69.7% *N* = 46	26.9% *N* = 7	One of the parents did not complete secondary education	74.2% *N* = 6	30.8% *N* = 7
Father did not complete post-secondary education	22.7% *N* = 15	11.5% *N* = 3	Mother did not complete post-secondary education	12.1% *N* = 8	26.9% *N* = 7	One of the parents did not complete post-secondary education	9.7% *N* = 10	26.9% *N* = 11
Father has some level of complete post-secondary education	34.8% *N* = 23	76.9% *N* = 20	Mother has some level of complete post-secondary education	18.2% *N* = 12	46.2% *N* = 12	Both parents have some level of complete post-secondary education	16.1% *N* = 62	42.3% *N* = 26

Chi-square *p = 0.001; **p = 0.001; ***p = 0.001

Education of the mother was related to the age of the student and the age of decision to study medicine. In the first case, median age of students tended to be lower among those with more educated mothers. On the contrary, decision to pursue a degree in medicine was taken earlier by students whose mothers did not complete post-secondary education. This was particularly obvious in male children of mothers that did not complete secondary education who decided to study medicine later than children of more educated mothers (Table 3).

**Table 3:** Distribution of education of mother by age of student and of decision to study medicine, stratified by sex

Education of the mother	Sex of students	Age of decision to study medicine*				Age of the student**			
		N	Mean	SD	Median	N	Mean	SD	Median
Mother did not complete secondary education	Male	27	17.2	6.0	17	40	23.4	1.9	23
	Female	5	10.8	1.6	12	6	24.0	2.2	24
	Total	32	16.2	6.0	16	46	23.5	1.9	23
Mother did not complete post-secondary education	Male	7	12.0	3.5	12	7	21.1	2.2	20
	Female	6	13.5	5.6	10.5	6	22.8	2.0	22.5
	Total	13	12.7	4.5	11	13	21.9	2.2	22
Mother has some level of complete post-secondary education	Male	10	13.5	3.2	13.5	11	22.3	1.6	22
	Female	7	13.1	2.3	12	10	22.7	2.8	22
	Total	17	13.4	2.8	13	21	22.5	2.2	22

ANOVA *p = 0.055; **p = 0.026

Students born in Bissau, who completed their primary education there, who wanted to practice in the capital city rather than somewhere else in the country and with family members in the health professions were more prone to have fathers with some sort of complete post-secondary education (Table 4).

**Table 4:** Relevant associations of education of father

Education of father	Place of birth of student is capital city vs somewhere else^*^(*n* = 43 vs 48)	Student completed primary education in capital city vs elsewhere^**^(*n* = 61 vs 30)	Student wants to practice medicine in capital city vs elsewhere^***^(*n* = 31 vs 61)	Students’ family members are health professionals vs not^#^ (*n* = 54 vs 38)
Father did not complete secondary education	16.3% vs 47.9% *N* = 7 vs 23	21.3% vs 58.6% *N* = 13 vs 17	25.8% vs 38.3% *N* = 8 vs 23	24.1% vs 69.2% *N* = 13 vs 18
Father did not complete post-secondary education	18.6% vs 20.8% *N* = 8 vs 10	23.0% vs 13.8% *N* = 14 vs 4	9.4% vs 25.0% *N* = 2 vs 16	18.5% vs 10.3% *N* = 10 vs 8
Father has some level of complete post-secondary education	65.1% vs 31.3% *N* = 28 vs 15	55.7% vs 27.6% *N* = 34 vs 9	65.6% vs 36.7% *N* = 21 vs 22	57.4% vs 20.5% *N* = 31 vs 12

Chi-square *p = 0.002; **p = 0.002; ***p = 0.024; ^#^*p* = 0.033

Combined education of parents was related with age of students: the parents of younger students completed secondary education but at least one of them did not complete post-secondary education and this was due to the interaction with the age of male rather than female students (Table 5).

**Table 5:** Relevant associations of combined education of parents

Combined education of both parents	Sex of students	N	Age of students		
			Mean	SD	Median
One of the parents did not complete secondary education	Male	38	23.3	1.8	23.0
	Female	7	23.7	2.1	22.0
	Total	45	23.4	1.9	23.0
One of the parents did not complete post-secondary education	Male	6	20.8	2.1	20.0
	Female	6	22.5	2.3	22.5
	Total	12	21.7	2.3	20.5
Both parents have some level of complete post-secondary education	Male	9	22.8	1.5	23.0
	Female	9	23.0	2.8	22.0
	Total	18	22.9	2.2	22.5

ANOVA *p* = 0.034

Students with parents with some level of complete post-secondary education considered less frequently a practice outside the capital city (Table 6).

**Table 6:** Distribution of combined education of parents with preferred location of future practice

Combined education of both parents	Student wants to practice	
	Capital city (*n* = 29)	Elsewhere (*n* = 59)
One of the parents did not complete secondary education	51.7% (*n* = 15)	66.1% (*n* = 39)
One of the parents did not complete post-secondary education	6.9% (*n* = 2)	6.9% (*n* = 11)
Both parents have some level of complete post-secondary education	41.4% (*n* = 12)	15.3% (*n* = 9)

Chi-square *p* = 0.018

## Discussion and conclusions

Our main interest in medical education is to study it as a health system intervention in order to contribute to health system’s strengthening in fragile states. This is discussed in the context of two frameworks: the labor market framework [35] and WHO’s health system strengthening framework [36].

The labor market framework extends the health workforce domain to include the educational sector, including pre-university education. This acknowledges that, as described from Cape Verde [37], Guiné-Bissau [38] and Brazil [39] students come ill-prepared from the secondary education sector to engage in university studies.

A recent review analyzed interventions that have been tried in higher- and middle-income countries to address health workforce training challenges. These concede the importance of the educational sector and include studies “on the themes of workforce planning, development of training school capacity, policies designed to attract students from underrepresented areas, and to under-favored specialties, as well as training financing policies and training partnerships” [40].

What is not acknowledged is that households and families may have an important role to influence decisions that lead children to train as health care workers, the age of those decisions and future practice expectations hence, contributing to strengthen the health system. Among the main reasons to choose medicine as a profession, family influence, particularly from relatives working as health professionals, is a major factor [30, 31, 33, 37, 41–46]. These same influences are felt for the choice of nursing studies [47].

The results of this analysis corroborate those previous findings and seems to partly relate that influence to the level of parental education. As reported in the literature [8, 13], our results confirm that the isolated effect of each parent is different as it is the combined education of both parents. As described elsewhere [8, 11], parental influence also seems to vary according to the sex of the offspring. The higher the education of the father, the stronger the urban background of the offspring. Level of education of parents is also important in relation to the decision to study medicine and the age of starting those studies. It is also an important influence as to expectation regarding place of future practice: the highest the educational level, particularly of the father, the highest the expectation for a future urban practice.

The results from this study also allow to identify the reproduction of gender-based inequalities in Guiné-Bissau that should be further analyzed in relation to broader policy issues. The reproduction of gender inequalities means the persistence of sex-driven social differences [48, 49]. In the case of medical students this is seen by the level of education of their parents (i.e., the higher weight of fathers with some levels of complete post-secondary education contrasts with the higher weight of mothers who did not complete secondary education) and the fact that the percentage of female students is decreasing in recent years (i.e., femininity in medical education lowered from 31 to 24% between 2007 and 2016 [33]). Not only is this decrease in counter cycle with what is observed in most (low income) countries, but also it makes clear the need of effective policies to overcome anachronistic gender-based differences in this country.

The reproduction of gender-based inequalities in medical education becomes even more relevant to highlight due to differences in the relevance of parents' educational level between male and female medical students: for male students the access to medical education is less dependent on the parents’ educational background than it is for female students. Saying differently: girls are more likely to progress in their studies in better educated families, while this relevance is less pronounced for boys. Although further studies on the resources that male students use to access medical schools are required, one key argument from these results is that the femininity of medicine in Guiné-Bissau depends directly on parents’ educational background. This is relevant for public policies aimed at strengthening better education for all as a mean to ensure inclusive and equitable quality education (sustainable development goal—SDG-4), achieve gender equality and empower women and girls (SDG 5), promote productive and decent work for all (SDG 8), and reduce inequalities within countries (SDG 10).

The recognition of the importance of family and parental education regarding choice of education and expectations regarding future practice are already reflected in recent adaptations of WHO’s health system strengthening framework that recognize the relevance of the household as another health system’s building block [50]. It is widely accredited that households are key “influencers of health for their families by making daily decisions regarding foods to eat, healthy practices in the home, and use of scarce resources for health care and better nutrition” [51]. Our data and that of others, as reported above, recognize that households are also important regarding future training and a future career in the health sector. This recognition warrants the integration of household characteristics and dynamics into HWFP frameworks. How to achieve that is the object of further scientific inquiry.

## Data Availability

The datasets used and/or analyzed during the current study are available from the corresponding author on reasonable request.

## Abbreviations

HWFP: Health workforce planning
SDG: Sustainable development goals
WHO: World Health Organization
